# EIT Outperforms Quantitative CT in Stratifying ARDS Severity After Lung Transplantation: A Retrospective Study

**DOI:** 10.1111/crj.70135

**Published:** 2025-11-13

**Authors:** Hui Jiang, Hu Zhao, Wei Cui, Xinxin Zhu, Ruoruo Yang, Yuqiang Wang, Xia Zheng

**Affiliations:** ^1^ Department of Critical Care Medicine The First Affiliated Hospital, Zhejiang University School of Medicine Hangzhou China; ^2^ Department of Critical Care Medicine Shandong University Affiliated Clinical Center for Public Health Jinan China; ^3^ Department of Critical Care Medicine and War Trauma Emergency Center The 903rd Hospital of the Joint Logistics Support Force Hangzhou China

## Abstract

**Background:**

Respiratory mechanics and gas exchange parameters are readily accessible indicators for evaluating ventilation in patients with acute respiratory distress syndrome (ARDS). Computed tomography (CT) is a widely used radiological imaging technique, while electrical impedance tomography (EIT) is a novel technique for pulmonary function monitoring developed in recent years. Studies suggest that EIT combined with quantitative CT holds unique value in assessing ventilation/perfusion (V/Q) function. Unlike classic ARDS, the pathophysiologic alterations in ARDS following lung transplantation are complex, and the specific mechanisms remain unclear. This study aims to explore the use of respiratory mechanics, gas exchange parameters, EIT, and quantitative CT for the multimodal assessment of V/Q function in post‐lung transplantation ARDS. We further aim to integrate visualizing dynamic imaging and regional quantitative lung analysis into this multimodal assessment system.

**Method:**

We retrospectively enrolled lung transplant recipients admitted to the intensive care unit who met the Berlin criteria for ARDS. Inclusion required an arterial partial pressure of oxygen to fraction of inspired oxygen ratio (P/F) ≤ 300 mmHg, with both EIT monitoring of V/Q and high‐resolution CT performed within 24 h of documented P/F ≤ 300 mmHg. Patient baseline characteristics, respiratory mechanics parameters, gas exchange parameters, EIT data, and CT images were collected. Subjects were stratified into two groups according to P/F values: a low P/F group (P/F < 200 mmHg) and a high P/F group (200 mmHg ≤ P/F ≤ 300 mmHg). Ventilation parameters derived from EIT included global inhomogeneity index (GI), center of ventilation (COV), and regional ventilation delay index (RVDI). Using hypertonic saline contrast‐enhanced EIT, we acquired V/Q parameters and calculated both global and regional EIT‐based dead space fraction (EIT‐Dead Space), intrapulmonary shunt fraction (EIT‐Shunt), and V/Q matching (EIT‐V/Q Match). Chest CT images were processed through a multitask learning U‐net‐based computer‐aided diagnostic model. This enabled semiautomated lung segmentation, identification of high‐density lesions, quantitative analysis, and three‐dimensional visualization. Pulmonary volumes, lesion volumes, and percentage lesion volumes (calculated as lung lesion volume divided by lung volume) were, respectively, quantified for the left and right lungs.

**Result:**

The study ultimately enrolled 21 lung transplant recipients with ARDS, comprising five patients in the low P/F group and 16 in the high P/F group. Among 21 patients, the low P/F group demonstrated significantly higher ventilation ratio (VR), RVDI, and EIT‐Dead Space, along with lower EIT‐V/Q match levels compared to the high P/F group, while no significant difference in EIT‐Shunt was observed between the two groups. EIT‐Dead Space showed substantial agreement with ventilator‐measured dead space fraction, and VR exhibited a significant positive correlation with EIT‐Dead Space. In the two groups, quantitative CT‐derived pulmonary metrics—including lung volume, lesion volume, and percentage lesion volume—showed no significant differences between the low and high P/F groups.

**Conclusion:**

Among lung transplant recipients with ARDS, the low P/F group demonstrated elevated VR, RVDI, and EIT‐Dead Space, alongside reduced EIT‐V/Q matching levels when compared to the high P/F group. Notably, no significant differences were found in the quantitative CT‐derived lesion volume parameters between the two groups.

## Graphical abstract

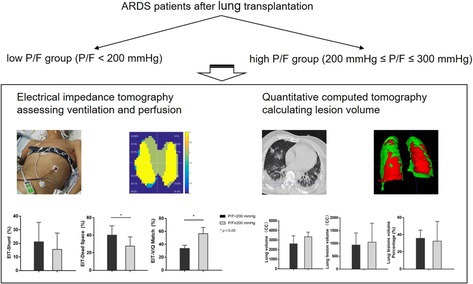

## 1 Introduction

Acute respiratory distress syndrome (ARDS), a life‐threatening form of acute respiratory failure with diverse etiologies, affects approximately 10% of critically ill patients in intensive care units (ICU) [1]. Notably, ARDS patients constitute 25% of all mechanically ventilated cases in the ICU, demonstrating mortality rates ranging from 30% to 40% [2]. The characteristic pathological triad includes diffuse alveolar damage, interstitial pulmonary edema, and hyaline membrane formation secondary to alveolar collapse. These structural alterations collectively contribute to impaired gas exchange through ventilation/perfusion (V/Q) mismatch, reduced lung compliance, and compromised oxygen diffusion capacity [3]. Particularly, the V/Q imbalance manifests as both increased dead space ventilation and intrapulmonary shunting, representing a critical pathophysiological determinant of refractory hypoxemia in ARDS [4]. Current evidence suggests that the severity of V/Q mismatch may serve as an independent prognostic indicator for mortality among mechanically ventilated ARDS patients [5]. The current therapeutic landscape for ARDS remains constrained by the absence of targeted pharmacological interventions, with lung‐protective ventilation strategies continuing to form the cornerstone of clinical management. This therapeutic challenge has been increasingly linked to the pathophysiologic heterogeneity of ARDS, prompting a paradigm shift in research focus toward elucidating its underlying mechanisms [6]. Notably, distinct from classic ARDS, post‐lung transplantation ARDS demonstrates unique patient‐specific factors and pathophysiologic heterogeneity, with their mechanistic foundations remaining incompletely understood [7]. These knowledge gaps underscore the clinical imperative for developing precise and multimodal V/Q assessment methodologies to characterize disease progression and facilitate personalized therapeutic approaches.

Lung transplantation is the only treatment option for end‐stage lung disease, and the most common indications include interstitial lung disease, advanced chronic obstructive pulmonary disease (COPD), emphysema caused by cystic fibrosis, and pulmonary arterial hypertension [8]. According to the International Society for Heart and Lung Transplantation Registry report, approximately 4500 adult lung transplants have been performed worldwide each year since 2017 [9, 10]. Despite the increasing number of cases of lung transplantation, various postoperative complications including primary graft dysfunction (PGD), acute rejection, chronic graft dysfunction, and infection remain the main obstacles to early and long‐term survival of patients after lung transplantation. PGD is an acute lung injury that occurs early after lung transplantation. Chest CT scans often show diffuse shadows in the lungs, often accompanied by ARDS, and are the most common cause of early death in patients after lung transplantation [10]. PGD has histopathological and clinical manifestations similar to ARDS, and its severity grading was similar to the Berlin definition of ARDS [11]. The V/Q mismatch caused by PGD or atelectasis is one of the main mechanisms of hypoxemia after lung transplantation. Pulmonary ventilation/perfusion scintigraphy and CT angiography can be used to evaluate lung V/Q mismatch, but the risk of nephrotoxicity and radiation damage from contrast agents needs to be considered. Therefore, patients after lung transplantation urgently need low‐risk and convenient lung V/Q monitoring methods.

Human gas exchange with the environment fundamentally depends on two interdependent processes: effective oxygenation and adequate ventilation. Oxygenation status is quantitatively assessed by the arterial partial pressure of oxygen to fraction of inspired oxygen ratio (P/F), which serves as the primary diagnostic and stratification criterion for ARDS. While ventilatory dysfunction can be monitored through physiological dead space fraction (V_D_/V_T_), multiple studies have demonstrated V_D_/V_T_'s value as an independent predictor of mortality in ARDS populations [12]. However, the clinical application of V_D_/V_T_ remains limited due to the lack of readily available monitoring techniques. Despite the ubiquitous presence of ventilatory impairment in ARDS, this parameter has not been incorporated into mainstream disease stratification systems [12]. Ventilatory ratio (VR), a readily obtainable bedside parameter, shows a strong correlation with V_D_/V_T_ measurements in ARDS patients [13]. Emerging evidence suggests that dead space predominance characterizes V/Q mismatch in COVID‐19‐associated ARDS [14].

Electrical impedance tomography (EIT), a functional pulmonary monitoring technique, operates through the application of safe alternating currents to thoracic surface electrodes to detect respiratory cycle‐induced variations in bioimpedance, with subsequent algorithmic reconstruction of regional ventilation distribution patterns [15]. Over the past decade, EIT has demonstrated considerable potential in critical respiratory care, particularly for ARDS management [16]. Clinical applications encompass recruitment monitoring, precision‐guided positive end‐expiratory pressure (PEEP) titration, and pulmonary perfusion assessment [17]. Compared with conventional pulmonary imaging modalities, EIT offers distinct advantages including liberation of patient transport requirements, radiation‐free operation, and bedside real‐time monitoring capabilities. The integration of EIT‐derived V/Q matching maps enables bedside evaluation of regional V/Q matching in critically ill COVID‐19 patients [18, 19]. Emerging evidence supports its diagnostic utility in detecting post‐lung transplantation V/Q mismatches secondary to vascular complications, providing a noninvasive approach for postoperative pulmonary function surveillance [20].

Computed tomography (CT) remains the cornerstone imaging modality in radiological diagnostics [21]. In ARDS management, high‐resolution chest CT plays a critical role in early disease detection and longitudinal monitoring. Initial CT findings in ARDS patients typically reveal diffuse bilateral ground‐glass opacities with gravitational distribution predominance, progressing to consolidation patterns in advanced stages [22]. Current clinical practice relies on subjective visual interpretation of CT imaging features for ARDS evaluation, while quantitative CT analysis—an emerging automated analytical approach—enables objective extraction and interpretation of radiomic biomarkers for diagnostic refinement and prognostication [23]. Evidence indicates that increased consolidation volume and lesion density on quantitative CT metrics correlate with disease severity in COVID‐19 patients [24]. Comparative physiological studies reveal significant disparities between COVID‐19‐associated ARDS and classic ARDS populations in CT‐derived parameters, oxygenation profiles, and respiratory mechanics characteristics.

## 2 Methods

### 2.1 Subjects

This study enrolled patients diagnosed with ARDS following lung transplantation. We retrospectively identified subjects who underwent lung transplantation between November 2021 and November 2022 at ICU in the First Affiliated Hospital, Zhejiang University School of Medicine, meeting the Berlin Definition criteria. The study received ethical approval from Clinical Research Ethics Committee of the above mentioned hospital (IIT2022017B‐R1). All transplant procedures utilized extracorporeal membrane oxygenation (ECMO) support with organs procured from brain‐dead donors. Inclusion criteria required (i) development of acute respiratory failure within 24‐h post‐ECMO decannulation, defined as P/F ≤ 300 mmHg; (ii) mechanical ventilation using pressure‐controlled mode with deep sedation ensuring absence of spontaneous respiratory effort; and (iii) completion of EIT‐based pulmonary V/Q monitoring within 24 h of acute respiratory failure diagnosis. Exclusion criteria comprised incomplete or suboptimal EIT data acquisition, absence of high‐resolution CT imaging within 24 h of meeting oxygenation criteria, and technical failures during quantitative CT analysis processing. We also excluded contraindications for EIT, such as spinal fractures, implantation of an implantable cardioverter defibrillator, and pacemaker.

### 2.2 Data Recording

This retrospective study utilized data extracted from electronic medical records with approval from the Clinical Research Supervisory Committee for informed consent exemption. Baseline characteristics recorded at enrollment comprised demographic parameters (age, gender, height, weight, and body mass index), comorbidities (hypertension and type 2 diabetes), biochemical markers (D‐dimer), RASS (Richmond agitation‐sedation scale), and APACHE II (acute physiology and chronic health evaluation II) at ICU admission. For lung transplant recipients, additional variables included primary pulmonary pathology, surgical site, and tobacco exposure history. EIT‐derived ventilation‐perfusion metrics were analyzed using raw monitoring data acquired within 24 h of enrollment, encompassing GI, COV, RVDI, and calculated parameters of dead space, shunt fraction, and ventilation‐perfusion matching. Concurrent ventilator parameters (ventilation mode, respiratory rate, tidal volume, minute ventilation, and positive end‐expiratory pressure) and arterial blood gas values (pH, partial pressures of oxygen/carbon dioxide, end‐tidal CO_2_, oxygen saturation, and fraction of inspired oxygen) were documented during the same 24‐h window. When multiple measurements existed, the dataset temporally closest to EIT acquisition was prioritized. Quantitative chest CT analysis was performed on images obtained within 24 h of enrollment (or nearest to EIT monitoring), calculating total lung volume, lobar volumetric measurements, and lesion burden quantification expressed as a percentage of pathological tissue relative to aerated parenchyma.

### 2.3 Variables

#### 2.3.1 VR

Minute volume (*MV*) is measured in milliliters per minute. Arterial partial pressure of carbon dioxide (*PaCO*_*2*_) is measured in mmHg. *PBW* stands for predicted body weight, measured in kilograms. The *PBW* calculation formula is as follows: Female, $\mathrm{PBW}=45.5+0.91\times \left(H-152.4\right)$, where H is the height, and the unit is cm. Male, $\mathrm{PBW}=50.0+0.91\times \left(H-152.4\right)$. The formula for calculating *VR* is as follows:

(1) $$\mathrm{VR}=\frac{\mathrm{MV}\times \mathrm{PaC}O_{2}}{\mathrm{PBW}\times 100\times 37.5}$$

#### 2.3.2 *C‐V*_*D*_ */V*_*T*_

*ETCO*_*2*_ is the end‐expiratory partial pressure of carbon dioxide, measured in mmHg. According to the Enghoff–Bohr equation, the dead space fraction *C*‐*V*_*D*_ */V*_*T*_ estimation formula for arterial blood gas estimation is as follows:

(2) $$C-V_{D}/V_{T}=\frac{{\text{PaCO}}_{2}-{\text{ETCO}}_{2}}{{\text{PaCO}}_{2}}$$

#### 2.3.3 Dynamic Lung Compliance

*V*_*TE*_ is the exhaled tidal volume, measured in mL. *Ppeak* is the peak airway pressure, measured in cmH_2_O. *PEEP* is positive end‐expiratory pressure, measured in cmH_2_O. It is required to measure under the condition of PCV and deep sedation without spontaneous breathing in the patient.

(3) $$\text{Cdyn}=\frac{V_{\mathrm{TE}}}{P_{\text{peak}}-\text{PEEP}}$$

V/Q assessment was performed using EIT with the PulmoVista 500 system (Dräger Medical). A 16‐electrode circumferential belt was positioned at the fourth intercostal space for continuous thoracic impedance monitoring at a 20‐Hz sampling frequency. Ventilatory parameters were acquired during standardized technical protocols: an expiratory hold maneuver (more than 8‐s duration) with concurrent central venous injection of 10 mL of 10% hypertonic saline for perfusion analysis [25]. Signal processing involved digital filtration via a 0.67‐Hz low‐pass filter to attenuate cardiogenic impedance oscillations.

Offline analysis was conducted using MATLAB R2015 (MathWorks Inc., Natick, MA) with custom algorithms defining ventilated and perfused regions based on 20% maximum pixel intensity thresholds. Three functional compartments were quantified: (i) dead space fraction (ventilation without perfusion), (ii) shunt fraction (perfusion without ventilation), and (iii) ventilation‐perfusion matched regions (concurrent ventilation and perfusion). Regional distributions were categorized as *R*_*V*_ (ventilation‐dominant), *R*_*P*_ (perfusion‐dominant), and *R*_*V+P*_ (matched regions) for pathophysiological correlation analysis.

#### 2.3.4 EIT‐Dead Space

The global dead space fraction is defined as the percentage of areas with only ventilation but no perfusion in the global system.

(4) $$\mathrm{EIT}-\text{Dead Space}=\frac{R_{V}}{\left(R_{V}+R_{P}+R_{V+P}\right)}\times 100\%$$

#### 2.3.5 EIT‐Shunt

The global pulmonary shunt fraction is defined as the percentage of areas with only perfusion but no ventilation that accounts for the global total.

(5) $$\mathrm{EIT}-\text{Shunt}=\frac{R_{P}}{\left(R_{V}+R_{P}+R_{V+P}\right)}\times 100\%$$

#### 2.3.6 EIT‐V/Q Match

The global ventilation/perfusion matching score (EIT‐V/Q Match) is defined as the percentage of areas with both perfusion and ventilation in the global system.

(6) $$\begin{array}{c} \mathrm{EIT}-V\;/\;Q\;\text{Match}=\frac{R_{V+P}}{\left(R_{V}+R_{P}+R_{V+P}\right)}\times 100\% \end{array}$$

### 2.4 Segmentation and Quantitative Analysis of CT Images

The chest CT scanning machines used in this study were all GE Revolution EVO. The patient lies flat on the scanning bed and begins scanning in a deep inhalation state, with the scanning range starting from the apex of the lung to the bottom of the lung. The scanning parameters are as follows: tube voltage is 120 kV, tube current is 260 mA, matrix is 512 × 512, scanning field is 350 mm × 350 mm, pitch is 1.375 mm, reconstruction layer thickness is 5 mm, and rotation speed is 0.35–1.0 s. After the scan is completed, the CT images are saved in the PACS imaging workstation. Export DICOM format files of images from the workstation for CT image segmentation, quantitative analysis, and lesion visualization.

We use computer‐aided diagnostic models for automatic identification and quantitative analysis of lung region segmentation and lesions. As the research subjects are patients with ARDS related to lung transplantation, the lesions identified by the model are patchy infiltrates and regional lung consolidation. The model is based on a multitask U‐net network for synchronous lung segmentation and lesion segmentation of input chest CT images [26]. The initialization and deduction of the model network used in this study are based on Dr. Pecker's cloud platform. The model has been pre‐trained with a large number of CT images in the early stage and has high reliability. The model demonstrated good accuracy in the initial 650 annotated chest CT scans. The average Dice similarity coefficient was 0.973 for the right lung, 0.985 for the left lung, and 0.864 for lesion segments. The model automatically calculates the density values within the region of interest in CT images and segments lung tissue completely according to anatomical structures [27]. The model divides lung tissue into left and right lungs, separated into lobes by lung fissures, and extracts and outputs quantitative imaging parameters of lesions including high grayscale ground‐glass opacities and consolidation opacities [28]. Including the volume of the entire lung and left and right lungs, the volume and quantity of lesions in the entire lung and left and right lungs, as well as the percentage of lesions in both lungs. The percentage of total lung lesion volume is the ratio of total lung lesion volume to total lung volume. The percentage of single‐lung lesion volume is the ratio of single‐lung lesion volume to total lung volume. The unilateral percentage of single‐lung lesions is the ratio of the volume of the single‐lung lesion to the volume of the single lung.

### 2.5 Statistical Analysis

We divided the study population into low P/F group (P/F < 200 mmHg) and high P/F group (200 mmHg ≤ P/F ≤ 300 mmHg) according to the P/F ratio. We performed statistical analysis using Stata MP and R 4.1.2 and used the *Shapiro–Wilk* test for the normality of quantitative data. If it conformed to a normal distribution, we expressed it as mean ± standard deviation and used *t*‐test for intergroup comparison; if it conformed to an abnormal distribution and was represented by the median (interquartile range), the *Wilcoxon* rank sum test was used for intergroup comparison. Categorical variables were expressed as examples (percentages), and *Fisher's* test was used for intergroup comparison. The test level for statistical analysis was 0.05; *p* < 0.05 was considered statistically significant. GraphPad Prism 7 was used for drawing statistical graphs.

## 3 Results

### 3.1 Demographic Baseline Characteristics

**FIGURE 1:**
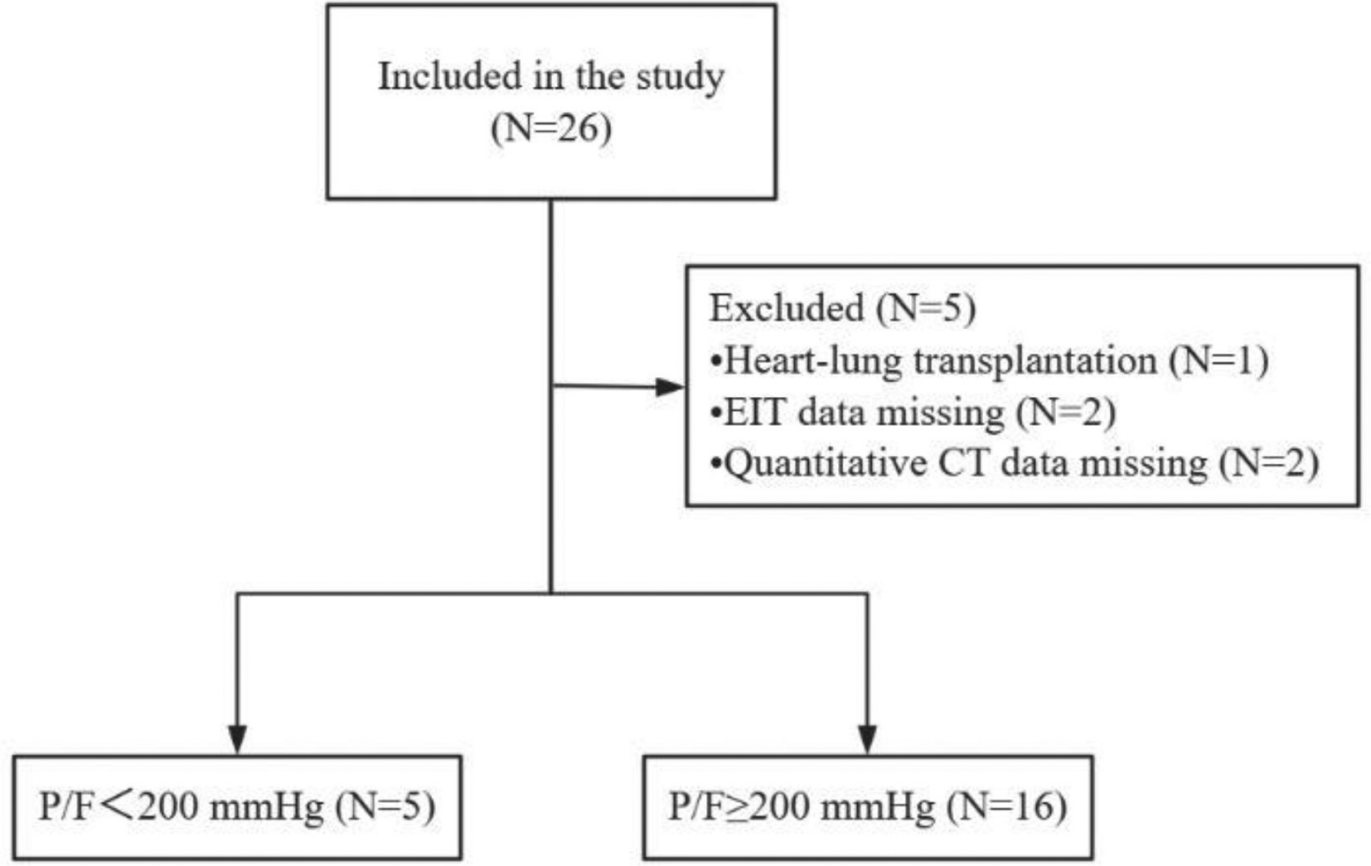
Flowchart of the study.

The study investigating multimodal pulmonary V/Q assessment in post‐lung transplantation ARDS patients enrolled 26 eligible subjects. With exclusions applied for combined cardiopulmonary transplantation (*n* = 1), missing EIT ventilation‐perfusion data (*n* = 2), and quantitative CT analysis errors (*n* = 2), the final cohort comprised 21 lung transplant recipients with ARDS. A flowchart detailing the study design is provided in Figure 1.

The final analysis included 21 patients stratified by oxygenation status: 5 in the low P/F group and 16 in the high P/F group. Demographic characteristics (Table 1) revealed a mean age of 53.00 ± 10.86 years with exclusively male predominance (100%) in the low P/F group, compared to 62.93 ± 6.52 years and 87.5% male representation (14/16) in the high P/F group. Surgical approaches comprised single‐lung transplantation in 10 patients and bilateral procedures in 11. Comorbidity profiles showed hypertension in 42.86% (9/21), diabetes mellitus in 9.52% (2/21), and smoking history in 42.86% (9/21). Primary pulmonary diagnoses included COPD (47.62%, 10/21) and interstitial lung disease (38.10%, 8/21). As detailed in Table 1, baseline characteristics demonstrated no significant intergroup differences except for age.

**TABLE 1:** Baseline characteristics of ARDS patients after lung transplantation.

	P/F < 200 mmHg	P/F ≥ 200 mmHg	*p*
	(*N* = 5)	(*N* = 16)	
Age (year)	53.00 ± 10.89	62.94 ± 6.53	0.047*
Female (*n*, %)	5 (100.00)	14 (87.50)	0.406
BMI (kg/m^2^)	21.14 ± 4.26	18.35 ± 2.92	0.110
Single‐lung transplantation (*n*, %)	2 (40.00)	8 (50.00)	0.696
Primary pulmonary disease (*n*, %)			
COPD	2 (40.00)	8 (50.00)	0.696
Interstitial lung disease	0 (0)	8 (50.00)	0.111
Past history (*n*, %)			
Hypertension	2 (40.00)	7 (43.75)	0.882
Diabetes	0 (0)	2 (12.50)	0.406
Smoking	4 (80.00)	5 (31.25)	0.119
D‐dimer (μg/L)	4114 (2671–6659)	5153 (1529–6603)	0.578
APACHE II (score)	22.00 ± 7.58	19.38 ± 7.40	0.499
RASS (score)	−3 (−4, −3)	−3 (−4, −3)	0.944

* *p* < 0.05.

### 3.2 Respiratory Mechanics and Gas Exchange Parameters

Respiratory mechanics and gas exchange parameters for lung transplant recipients developing ARDS are presented in Table 2. Comparative analysis revealed no significant intergroup differences in inspiratory pressure, dynamic compliance (Cdyn), tidal volume per predicted body weight (TV/PBW), PEEP, respiratory rate, or fraction of inspired oxygen (FiO_2_). Quantitatively, TV/PBW measured 6.47 ± 0.94 mL/kg in the low P/F group versus 6.64 ± 1.11 mL/kg in the high P/F group. Oxygenation profiles demonstrated clinically relevant disparities: the low P/F group exhibited lower peripheral oxygen saturation (SpO_2_) and PaO_2_, coupled with elevated arterial carbon dioxide tension (PaCO_2_), reflecting more pronounced hypoxemia compared to the high P/F group. Acid‐base equilibrium parameters showed comparable values between the two groups, with pH levels of 7.40 ± 0.09 (low P/F group) versus 7.41 ± 0.06 (high P/F group) (*p* = 0.934). End‐tidal carbon dioxide (ETCO_2_) measurements were similarly nonsignificant at 38.0 (36.0–46.0) mmHg versus 31.0 (29.0–35.5) mmHg (*p* = 0.115).

**TABLE 2:** Respiratory mechanics and gas exchange parameters of ARDS patients after lung transplantation.

	P/F < 200 mmHg	P/F ≥ 200 mmHg	*p*
	(*n* = 5)	(*n* = 16)	
Inspiratory pressure (cmH_2_O)	23.20 ± 3.96	22.75 ± 3.51	0.967
PEEP (cmH_2_O)	7.52 ± 2.09	7.38 ± 1.53	0.869
Cdyn (mL/cmH_2_O)	30.64 ± 4.64	27.78 ± 8.88	0.551
TV/PBW (mL/kg)	6.47 ± 0.94	6.64 ± 1.11	0.761
Respiratory rate (times/min)	17.40 ± 2.41	18.94 ± 4.25	0.455
FiO_2_ (%)	40 (30–40)	33 (30–35)	0.228
SpO_2_ (%)	93.40 ± 2.61	96.44 ± 1.97	0.006*
PaO_2_ (mmHg)	66.0 (59.0–73.0)	84.5 (78.0–88.5)	0.009*
PaCO_2_ (mmHg)	44.0 (43.0–50.0)	39.5 (34.5–41.5)	0.008*
PH	7.40 ± 0.09	7.41 ± 0.06	0.934
ETCO_2_ (mmHg)	38.0 (36.0–46.0)	31.0 (29.0–35.5)	0.115
V‐V_D_/V_T_ (%)	29.40 ± 5.55	21.19 ± 6.87	0.725
C‐V_D_/V_T_ (%)	20.92 ± 14.42	17.76 ± 10.06	0.585
VR	1.53 ± 0.22	1.19 ± 0.24	0.006*

* *p* < 0.05.

Ventilator‐derived dead space fraction (V‐V_D_/V_T_) presented in Table 2 was acquired through *Medibus* interface data transmission (the data connection between the ventilator and EIT machine) from ventilator‐integrated measurements. C‐V_D_/V_T_ was determined using arterial blood gas parameters via Equation (2) as previously described. The low P/F group demonstrated V‐V_D_/V_T_ values of 29.40 ± 5.55 compared to 21.19 ± 6.87 in the high P/F group, though these differences lacked statistical significance. Similarly, C‐V_D_/V_T_ showed comparable values between the two groups (20.92 ± 14.42 vs. 17.76 ± 10.06, *p* = 0.585). A marked intergroup disparity emerged in VR, with the low P/F group exhibiting significantly elevated values (1.53 ± 0.22) relative to the high P/F group (1.19 ± 0.24, *p* = 0.006 < 0.05), indicating impaired ventilation efficiency in the former group.

### 3.3 Pulmonary V/Q Assessment Based on EIT

**FIGURE 2:**
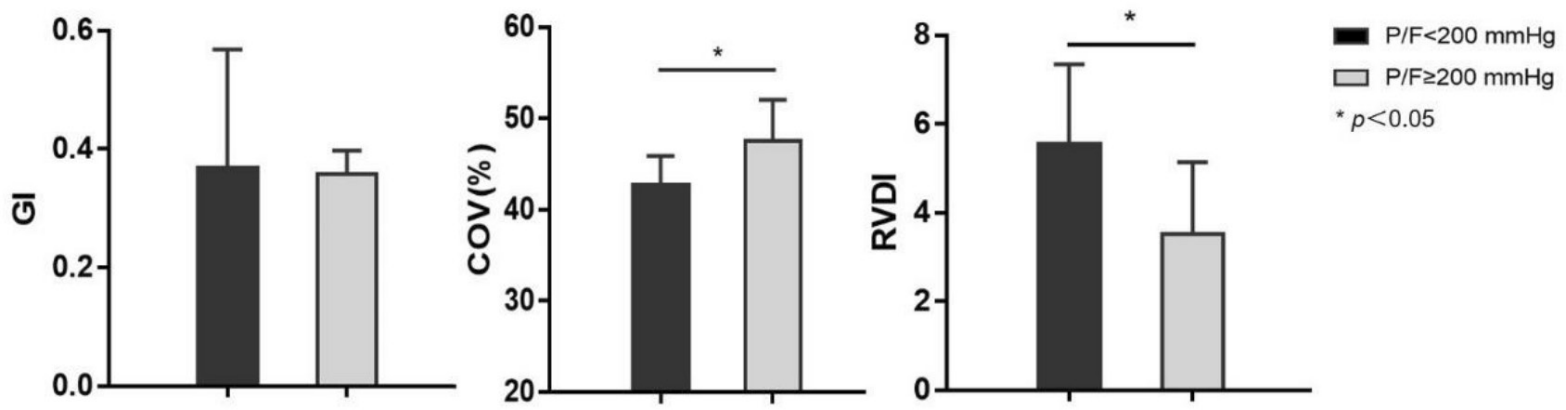
GI, COV, and RVDI in ARDS patients after lung transplantation in low P/F and high P/F groups.

As shown in Figure 2, in lung transplant recipients with ARDS, GI demonstrated comparable values between the low P/F group (median 0.37, IQR 0.35–0.46) and the high P/F group (median 0.35, IQR 0.33–0.38), with no statistically significant difference (*p* = 0.248). Contrastingly, COV was significantly elevated in the low P/F group (42.74% ± 3.14%) compared to the high P/F group (47.53% ± 4.52%, *p* = 0.041), indicating greater regional ventilation heterogeneity. The analysis of RVDI revealed a similar pattern, with the low P/F group exhibiting higher values (median 5.54, IQR 4.29–5.87) versus the high P/F group (median 3.49, IQR 2.45–4.24, *p* = 0.039), suggesting impaired synchrony of ventilation distribution in patients with severe hypoxemia.

According to the horizontal stratification method, the interface between lung ventilation and perfusion imaging displayed by EIT is divided into four regions of interest (ROI) from ventral to dorsal: most nondependent (ROI L1), nondependent (ROI L2), second dependent (ROI L3), and most dependent (ROI L4). The ventilation and perfusion distribution of patients in the low P/F group and the high P/F group at ROI L1, ROI L2, ROI L3, and ROI L4 are shown in Figures S1 and 3. It was found that the two groups were concentrated in ROI L2 and ROI L3 for ventilation and perfusion distribution. ROI L1 and ROI L4 have less distribution in ventilation and perfusion. There was no statistically significant difference in the distribution of the four ROIs between the low P/F group and the high P/F group patients.

**FIGURE 3:**
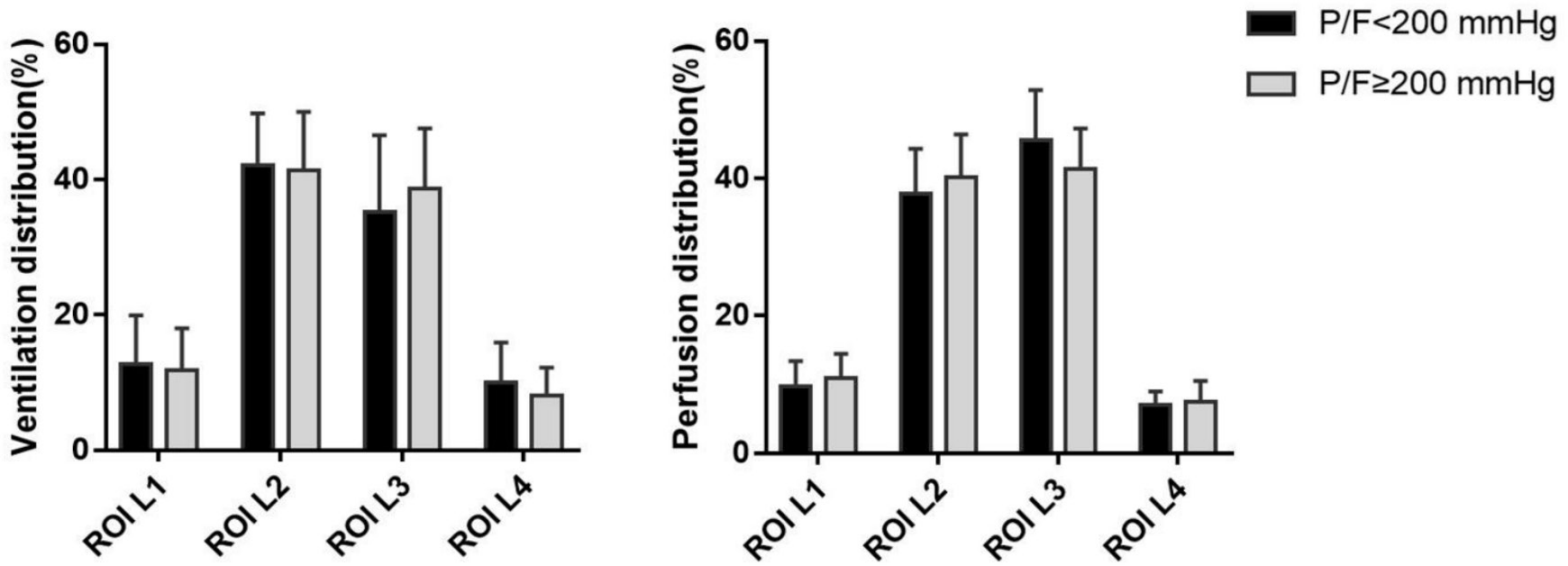
Ventilation and perfusion distribution in ARDS patients after lung transplantation in low P/F and high P/F group.

**FIGURE 4:**
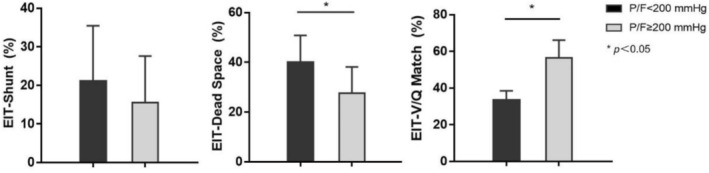
EIT lung V/Q parameter in ARDS patients after lung transplantation in low P/F and high P/F groups.

Comparative analysis of EIT parameters between two groups revealed no statistically significant difference in EIT‐derived shunt fraction (EIT‐Shunt: 21.12% ± 14.33% vs. 15.58% ± 12.04%, *p* = 0.620). However, the low P/F group demonstrated significantly elevated dead space fraction (EIT‐Dead Space: 40.10% ± 10.71% vs. 27.50% ± 10.62%, *p* = 0.032) and reduced ventilation‐perfusion matching (EIT‐V/Q Match: 33.58% ± 4.95% vs. 56.45% ± 9.71%, *p* = 0.002) compared to the high P/F group (Figure 4). These findings suggest that post‐lung transplant ARDS patients with low P/F exhibit greater physiological dead space and impaired ventilation‐perfusion coordination relative to those with high P/F profiles.

### 3.4 Consistency Verification of V‐V_D_/V_T_, C‐V_D_/V_T_, and EIT Dead Space

**FIGURE 5:**
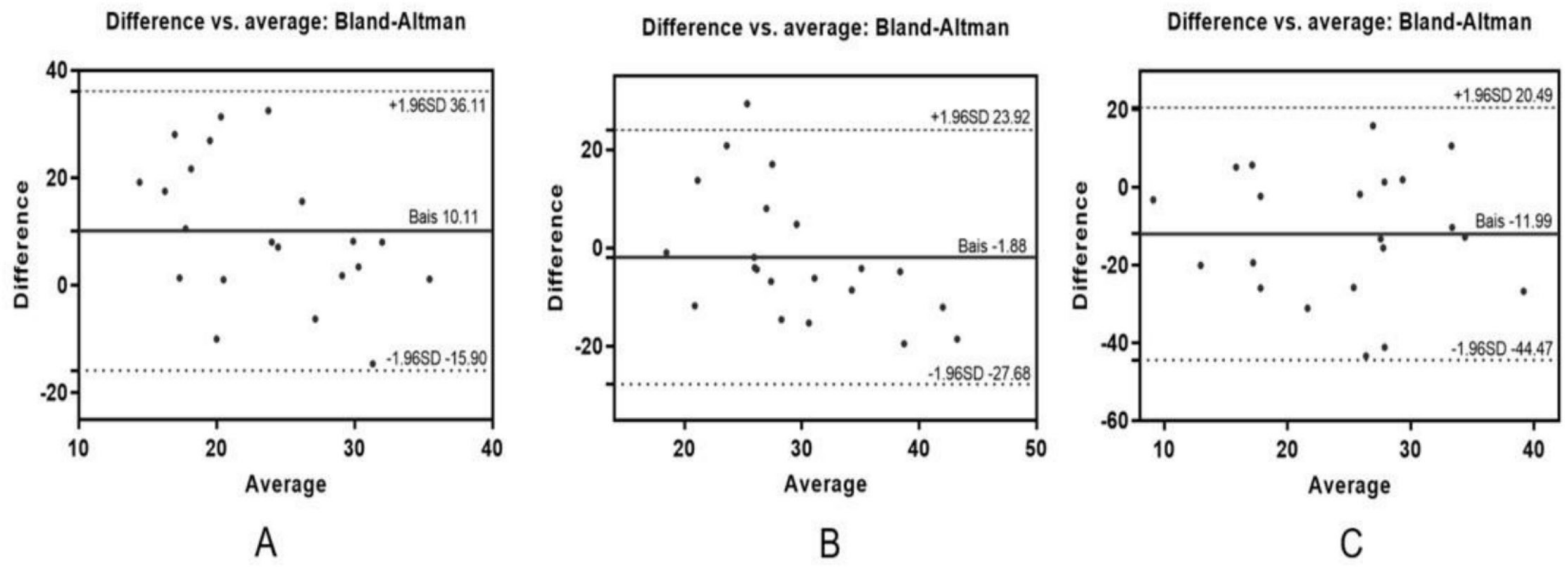
Bland–Altman plot of the mean difference between three dead space calculation methods.

Methodological agreement among three dead space quantification approaches was assessed using Bland–Altman analysis (Figures 5 and S2; Table S1). V‐V_D_/V_T_ demonstrated statistically significant divergence from C‐V_D_/V_T_ based on arterial blood gas analysis, with a mean difference of 10.108 (paired *t*‐test, *p* = 0.05). Similarly, a significant discrepancy was observed between C‐V_D_/V_T_ and EIT‐Dead Space, showing a mean difference of −11.990 (*p* = 0.05). In contrast, comparative analysis of ventilator‐measured V‐V_D_/V_T_ and EIT‐Dead Space revealed no statistically significant mean difference (−1.882, paired *t*‐test *p* = 0.52). All measurement pairs resided within the 95% confidence interval limits, confirming acceptable agreement between these two methodologies.

Correlation analyses were performed between VR and V‐V_D_/V_T_, VR and C‐V_D_/V_T_, as well as VR and EIT‐Dead Space using Pearson's correlation coefficient to evaluate the strength of associations. The results revealed a correlation coefficient of 0.376 (*p* = 0.093) between VR and V‐V_D_/V_T_, indicating no statistically significant association between VR and ventilator‐estimated dead space fraction. The correlation coefficient between VR and C‐V_D_/V_T_ was 0.025 (*p* = 0.914), approaching a null value with a nonsignificant *p*‐value, demonstrating no correlation between VR and blood gas parameter‐calculated dead space fraction. In contrast, VR exhibited a significant positive correlation with EIT‐Dead Space, evidenced by a correlation coefficient of 0.531 that reached statistical significance at the 0.05 level.

### 3.5 Evaluation of Lung Lesions Based on Quantitative CT

Quantitative CT analysis of post‐lung transplant ARDS patients revealed no significant differences between the low P/F and high P/F groups (Table 3 and Figure S3). Total lung volumes were 3537.28 ± 770.33 cm^3^ in the low P/F group versus 3212.34 ± 1269.74 cm^3^ in high P/F group (*p* = 0.509). The total lesion volume measured 765.38 ± 304.24 cm^3^ in low P/F group compared to 611.85 ± 291.57 cm^3^ in high P/F group (*p* = 0.283). The percentage of total lesion volume relative to whole lung volume was 22.42% ± 8.74% in the low P/F group and 22.85% ± 13.51% in the high P/F group (*p* = 0.901). These results demonstrate that quantitative CT‐derived parameters—including total lung volume, total lesion volume, and lesion volume percentage—showed no statistically significant differences between low P/F and high P/F groups in ARDS patients following lung transplantation.

**TABLE 3:** CT quantitative parameter table for ARDS patients after lung transplantation.

Category	P/F < 200 mmHg	P/F ≥ 200 mmHg	*p*
	(*n* = 5)	(*n* = 16)	
Left lung volume (CC)	1638.00 ± 439.76	1411.22 ± 666.18	0.283
Left lung lesion volume (CC)	371.88 ± 212.77	356.33 ± 190.32	1
Right lung volume (CC)	1899.24 ± 442.91	1801.13 ± 718.15	0.563
Right lung lesion volume (CC)	393.50 ± 105.17	255.52 ± 135.85	0.058
Percentage of left lung lesion volume (%)	10.64 ± 5.32	12.98 ± 7.96	0.620
Percentage of right lung lesion volume (%)	11.77 ± 4.36	9.87 ± 6.30	0.457
Percentage of left lung lesion in unilateral lung (%)	22.32 ± 9.69	31.63 ± 20.79	0.509
Percentage of right lung lesion in unilateral lung (%)	22.24 ± 8.50	17.76 ± 11.87	0.364

## 4 Discussion

In patients with ARDS after lung transplantation, pulmonary injury induces complex pathophysiological alterations characterized by impaired gas exchange and reduced pulmonary compliance. Our study revealed comparable dynamic compliance values between the low P/F and high P/F groups (30.64 ± 4.64 vs. 27.78 ± 8.88 mL/cmH_2_O, *p* = 0.551). The interpretation of pulmonary compliance in post‐transplant patients requires cautious consideration due to multifactorial determinants. Notably, inherent compliance exists between native and transplanted lungs in single‐lung recipients [29], while bilateral transplantation outcomes may correlate with donor organ quality. Therefore, the above results are not sufficient to reveal valuable conclusions related to lung transplant compliance. V_D_/V_T_ serves as a critical physiological parameter for assessing V/Q mismatch in ARDS, with established prognostic value for mortality risk as demonstrated in prior studies [30]. While multiple V_D_/V_T_ estimation methodologies have evolved since their initial conceptualization [31], our investigation in post‐lung transplant ARDS patients revealed distinct concordance patterns: EIT‐derived dead space measurements showed significant agreement with V‐V_D_/V_T_, but not with the C‐V_D_/V_T_ method based on arterial blood gas parameters. The V‐V_D_/V_T_ quantification in this study utilized continuous analysis of end‐tidal CO_2_ waveforms and tidal volume dynamics. However, the C‐V_D_/V_T_ methodology exhibited inherent measurement instability, likely attributable to physiological fluctuations in arterial blood gas parameters. These findings underscore the necessity for standardized reference criteria and mechanistic investigations into the pathophysiological implications of different V_D_/V_T_ measurement approaches in pulmonary V/Q assessment. VR, a novel bedside‐accessible metric calculated from routine blood gas parameters [32], has demonstrated significant correlations with V_D_/V_T_ in ARDS patients, establishing its utility as a surrogate marker for ventilation‐perfusion mismatch [33]. In post‐lung transplant populations, our study revealed significant positive associations between VR and EIT‐derived dead space measurements. This physiological parameter exhibits progressive elevation with ARDS severity, particularly evident in patients with profound hypoxemia, where elevated VR values correlate with advanced disease stages [12]. Our observations align with these pathophysiological insights, suggesting VR's potential role in quantifying ventilation impairment severity across ARDS progression.

RVDI, a quantitative EIT parameter reflecting temporal ventilation heterogeneity, demonstrated significant elevation in the low P/F group compared to the high P/F group, with concomitant reductions in COV—a metric quantifying the proportion of dorsal pulmonary ventilation to total ventilation. These findings may suggest increased temporal ventilation asynchrony and dorsal ventilation predominance in moderate‐to‐severe ARDS following lung transplantation. We also observed that the proportion of the gravity‐dependent region in the low P/F group seemed to be smaller than that in the high P/F group, even though this difference was not statistically significant. To be honest, this inconsistency may be related to the limited number of included cases, so we failed to draw a definite argument. Furthermore, the low P/F group exhibited elevated EIT‐Dead Space values and diminished EIT‐V/Q Match, though no significant intergroup differences emerged in EIT‐Shunt. EIT‐based V/Q monitoring provides diagnostic utility in this population, enabling simultaneous evaluation of ARDS‐induced pathophysiological alterations and surveillance of transplant‐specific vascular complications. This is exemplified by Zarantonello et al.'s pioneering application of EIT‐derived V/Q parameters for monitoring post‐transplant pulmonary artery stenosis [20]. Compared to conventional diagnostic modalities like ventilation/perfusion scintigraphy and CT angiography, EIT offers distinct advantages including radiation‐free operation, non‐invasiveness, and real‐time bedside monitoring capabilities. Crucial to emphasize is the algorithmic derivation of V/Q matching, which quantifies regional ventilation‐perfusion distribution ratios relative to global pulmonary patterns through EIT reconstruction. This computational metric fundamentally differs from the classical physiological V/Q ratio defined as alveolar ventilation to pulmonary blood flow per unit time. Clinicians should be cognizant of this technical distinction to avoid misinterpretation of EIT‐derived parameters as direct equivalents to conventional gas exchange indices.

In this study, quantitative CT parameters assessing pulmonary consolidation volumes failed to demonstrate statistically significant differences between low P/F and high P/F groups. While low P/F correlated with elevated EIT‐derived dead space values, these findings were not accompanied by proportional increases in radiologically quantified lesion volumes or consolidation percentages. We speculate that this may be due to the following reasons. Firstly, ARDS exhibits patient heterogeneity. In clinical practice, we have observed that some ARDS patients present with large areas of lung consolidation on CT images, but EIT's lung V/Q combined imaging shows good V/Q matching. The clinical manifestations of hypoxemia in some patients are severe, but CT scans reveal smaller lung lesions, and the reason is still unknown. In fact, the factors that can explain the improvement of oxygenation are complex and not limited to the improvement of gas exchange. The increase or decrease in the volume of pulmonary infiltration in quantitative CT may not necessarily predict the deterioration or improvement of oxygenation, and there may also be inconsistencies between clinical symptoms and chest CT imaging findings. Secondly, the divergent pathophysiological representations between EIT and quantitative CT stem from fundamental methodological differences. EIT‐derived V/Q Match is calculated as the proportion of regional ventilation‐perfusion distribution relative to the global impedance‐varying domain within the electrode belt plane [34], where “global” specifically refers to the anatomical cross‐section generating detectable impedance changes [35]. Crucially, regions without impedance variation—potentially representing pleural effusions, localized pneumothorax, or subcutaneous emphysema—remain undetected by this technology [36]. In post‐lung transplant ARDS patients with prolonged supine positioning, gravity‐dependent consolidation zones exhibiting combined alveolar collapse and vascular obstruction create functionally silent regions that evade EIT quantification. This discrepancy may become clinically significant when physiologically silent zones occupy substantial anatomical volumes, potentially explaining observed mismatches between EIT‐derived V/Q parameters and CT morphological findings. The complementary value of these modalities is exemplified in Claude et al.'s seminal case documenting combined EIT‐CT monitoring in a bilateral lung transplant recipient [37]. While CT revealed extensive left pulmonary consolidation with right lung hyperlucency, EIT demonstrated near‐complete right lung functional collapse with preserved left lung ventilation. This striking divergence underscores EIT's unique capacity to provide functional insights beyond CT's structural visualization, particularly critical for differentiating native versus graft lung pathophysiology in transplant recipients.

Furthermore, we observed discrepancies between CT and EIT in assessing disease severity, which may be partly explained by the use of planar EIT rather than spatially resolved three‐dimensional EIT (3D‐EIT). 3D‐EIT involves placing two horizontal electrode belts at the level of the 3rd and 5th intercostal spaces and relying on computer algorithms to reconstruct spatial images. In theory, 3D‐EIT can provide more spatial resolution information compared to conventional EIT. This advantage is illustrated in a case reported by Dr. He et al., wherein 3D‐EIT detected a right pulmonary artery embolism in a VA‐ECMO patient with no significant CT abnormalities, highlighting its potential for improved diagnostic accuracy [38]. Conventional EIT does not provide spatial resolution along coronal or sagittal planes, limiting its ability to capture heterogeneous V/Q patterns often seen in pathological lung conditions. In contrast, 3D‐EIT may offer more detailed and individualized lung assessment capabilities. Additionally, conductivity changes caused by extraplanar thoracic lesions may further confound 2D‐EIT interpretations.

The application of EIT in lung transplantation has seen growth across various clinical aspects, including donor lung management, functional pulmonary imaging, recruitment maneuvers guidance, ventilation heterogeneity monitoring, postoperative complications detection, and lung perfusion assessment [39]. The optimal mechanical ventilation strategy for lung transplantation has not been fully established, with the prevailing approach favoring a lung‐protective strategy [40]. Individualized PEEP titration following transplantation is desirable to mitigate ventilator‐induced lung injury and minimize pulmonary complications [41]. Notably, EIT‐guided PEEP titration offers a potential means to personalize ventilation settings at the bedside for transplant recipients [42]. In addition, there are other pulmonary imaging techniques to monitor the V/Q match in lung transplantation. Single‐photon emission computed tomography (SPECT), frequently combined with CT (SPECT/CT), is a nuclear medicine imaging technique that provides three‐dimensional functional information [43]. In lung transplantation, SPECT has been primarily utilized for lung perfusion scintigraphy, playing a crucial role in assessing graft function and detecting postoperative complications [44, 45, 46]. Nakashima et al. employed SPECT to evaluate V/Q characteristics following lung transplantation. By analyzing the functional volume derived from V/Q SPECT/CT and the morphological volume obtained from CT imaging, they proposed a functional volume‐to‐morphological volume ratio. This semiquantitative metric facilitates the assessment of pulmonary ventilation and perfusion functions, demonstrating particular utility in diagnosing bronchiolitis obliterans syndrome after transplantation [47]. Similarly, Mohammad et al. reported the application of V/Q SPECT/CT for detecting clinically silent pulmonary thromboembolism in double‐lung transplant recipients [48]. Besides, Cheng et al. innovatively combined EIT and positron emission tomography to attempt to establish multimodal imaging evidence linking regional airway obstruction with impaired drug deposition [49]. We can boldly speculate that SPECT can also be combined with EIT to explore V/Q information not only in lung transplantation but also in other respiratory pathophysiological states.

This study has several noteworthy limitations. The modest sample size reflects inherent challenges in patient recruitment within China's developing lung transplant ecosystem, where fewer than 50 centers currently hold transplantation accreditation and only nine institutions reported annual case volumes exceeding 10 procedures in 2019 (data from China Organ Transplant Registry). While our cohort surpasses the sample size of Ramanathan et al.'s seminal EIT study in lung recipients (*n* = 6) [50], statistical power remains constrained for detecting subtle pathophysiological differences. These limitations are compounded by the single‐center design and heterogeneity in postoperative management protocols across institutions. Future multicenter collaborations incorporating standardized EIT acquisition protocols could enhance the generalizability of findings while addressing the unique logistical challenges of transplant‐related ARDS research.

## 5 Conclusion

Our study found that among lung transplant recipients with ARDS, the low P/F group demonstrated elevated VR, RVDI, and EIT‐Dead Space, alongside reduced EIT‐V/Q matching levels when compared to the high P/F group. Notably, no significant differences were found in the quantitative CT‐derived lesion volume parameters between the two groups. It is worth noting that no significant differences were found in the quantitative CT‐derived lesion volume parameters between the two groups. In addition, we also discussed the clinical concerns derived from the above findings and the application of other lung function imaging in lung transplantation.

## Author Contributions

Designed study: Hui Jiang, Xia Zheng. Performed study: Hui Jiang, Yuqiang Wang. Collected data: Hui Jiang, Hu Zhao, Wei Cui. Analyzed data: Hu Zhao, Xinxin Zhu, Ruoruo Yang. Wrote the paper: Hui Jiang, Hu Zhao.

## Ethics Statement

The study received ethical approval from Clinical Research Ethics Committee of the First Affiliated Hospital, Zhejiang University School of Medicine (IIT2022017B‐R1). The information collected in this study has obtained written consent from the patient representative.

## Conflicts of Interest

The authors declare no conflicts of interest.

## Supporting information


**Figure S1:** Diagram of horizontal layering in EIT images.
**Figure S2:** Line graph of mean and 95% confidence interval of three dead cavity calculation methods.
**Table S1:** Bland–Altman analysis tests for V‐V_D_/V_T_, C‐V_D_/V_T_, and EIT Dead Space.
**Figure S3:** CT quantitative parameter bar chart of ARDS patients after lung transplantation in low P/F and high P/F groups.

## Data Availability

The data that support the findings of this study are available on request from the corresponding author. The data are not publicly available due to privacy or ethical restrictions.
